# Language and Culture Backgrounds of Mothers and Child Development: A Nationwide Study on the Incidence of Developmental Delays in Children Born to Immigrant Mothers in Taiwan

**DOI:** 10.3389/fpubh.2021.646444

**Published:** 2021-08-24

**Authors:** Yen-Cheng Tseng, Der-Chung Lai, How-Ran Guo

**Affiliations:** ^1^Language Education Center and Department of Tourism, Food, and Beverage Management, Chang Jung Christian University, Tainan, Taiwan; ^2^Department of Physical Medicine and Rehabilitation, Ditmanson Medical Foundation Chiayi Christian Hospital, Chiayi, Taiwan; ^3^Department of Nursing, Chung-Jen Junior College of Nursing, Health Sciences and Management, Chiayi, Taiwan; ^4^Department of Occupational and Environmental Medicine, National Cheng Kung University Hospital, Tainan, Taiwan; ^5^Occupational Safety, Health and Medicine Research Center, National Cheng Kung University, Tainan, Taiwan; ^6^Department of Environmental and Occupational Health, National Cheng Kung University Hospital, Tainan, Taiwan

**Keywords:** culture, developmental delays, immigrant mother, language, Taiwan

## Abstract

**Background:** Transnational marriages are common as a result of globalization, and immigrant mothers face various degrees of differences in language and culture backgrounds. Mothers have great influences on the development of their children, but the effects of immigrant mothers' language and culture backgrounds on developmental delays (DD) are seldom studied. To evaluate the potential effects of immigrant mothers' language and culture backgrounds on DD of their children, we conducted a nationwide study in Taiwan.

**Methods:** We analyzed the data from the national registry of DD in Taiwan from 2010 to 2013 and compared the incidence of DD in young children born to mothers from China, Vietnam, and Indonesia, where most of the immigrant mothers in Taiwan come from. Amongst the three countries, China is the closest to Taiwan in terms of language and culture, followed by Vietnam, and then Indonesia.

**Results:** We identified 4,604 patients of DD in children under 7 years old. The incidence rates showed an increasing trend among children born to mothers from China, Vietnam, and Indonesia (*p* < 0.01 in all years). Using children born to mothers from Vietnam, whose incidence rate of DD was in the middle amongst the three groups, as the reference, we found the incidence rate ratios in children born to mothers from China ranged from 0.65 to 0.73, and those in children born to mothers from Indonesia ranged from 1.04 to 1.26.

**Conclusions:** The findings support the important role of mothers' language and culture backgrounds in the development of children.

## Introduction

Developmental delays (DD) in children are common all over the world and thus constitute an important issue in childhood health (1, 2). A child's development is affected by a complex mix of factors, and maternal factors play an important role. Among these maternal factors, the biological factors include genetics, behaviors (such as abuse of drugs or alcohol), health, and exposure to environmental toxins. Maternal social factors are also very important, including maternal education, teenage mother, social or financial support, etc. (3–5). Mothers' language and culture backgrounds could be considered as social factors, but their effects on child development were seldom studied. In terms of mothers' languages, a study examined the relation of maternal language to language delay of children and found mothers of language-delayed children used more irrelevant language than did mothers of children without language delay (6). In addition, a study in Taiwan that included 61 children born to mothers from China and Vietnam found that maternal language ability was associated with child development (7).

Mothers' culture backgrounds may also affect children's development (8, 9). For example, some indigenous cultures allow drinking alcohol during pregnancy, which may increase the risk for fetal alcohol syndrome (10). In fact, parenting knowledge could be regarded as a part of culture (11, 12) and may affect mothers' decisions on their children's care, which in turn affect children's development. A study investigated parenting knowledge in Japanese and South American immigrant mothers and found that immigrant mothers had significantly less parenting knowledge than multigenerational US mothers (12). In addition, mothers of foreign nationalities might also face conflicts about passing on their cultural identities to their children, which may affect the development of the children (4).

With globalization, transnational marriages are increasing globally. In Taiwan, for example, they have been increasing in the past two decades and reached the peak in 2003, when they constituted 31.86% of all marriages in the country (13). Whereas the proportion started to decrease in 2004, it was as high as 13.77% in 2016 (14). The majority of transnational marriages in Taiwan are those between native men and foreign women, and most immigrant mothers are from China and Southeast Asia, particularly Vietnam and Indonesia (15). Of the newborns in Taiwan in 2016, 6.19% were from mothers of foreign nationalities (15). Immigrant mothers face various degrees of differences in language and culture backgrounds in the new environment. Amongst the three, China is the closest to Taiwan in terms of language and culture, followed by Vietnam, and then Indonesia (16, 17).

While an immigrant mothers' language and culture backgrounds may have effects on her children's development, using “developmental delay” combining “immigrant mother” or “foreign mother” as the keywords to search literature indexed in PubMed, we could not find any reports on this issue. In Taiwan, the government is required to establish a reporting system for children with DD by the Children Welfare Law (18), and the government maintains a registry of cases from the whole nation. The registry data constitute a rarely available resource for studying DD at a national level and have been used to study the epidemiology of DD in Taiwan (14, 19, 20). To assess the associations between immigrant mothers' language and culture backgrounds and the incidence of DD in their children, we conducted a study using the data obtained from the national registry. We made comparisons amongst children of immigrant mothers, so that certain degree of variations in language and culture backgrounds in the study population can be ensured, while the effects of factors associated with immigration (decision on leaving the home country, process of immigration, adaptation to the new environment, etc.) can be minimized.

## Methods

### Case Definition

The Enforcement Rules of the Children and Youth Welfare Law (21) defined “developmental delays” as “allegedly or expected abnormal development in respect of cognitive development, physiological development, language and communication development, psycho-social development or self-governing skills that have been judged and confirmed by the accredited medical institutes under health authority.” Under the regulations in Taiwan, parents and welfare, education, and medical institutions can report suspected cases to Early Intervention Reporting and Referral Centers (EIRRCs), but EIRRCs register only the cases certified by accredited hospitals or trained social workers employed by EIRRCs (22). The hospitals are accredited by the Ministry of Health and Welfare, and the social workers generally use the 2nd Version of Taipei City Developmental Checklist for Preschoolers as the tool to conduct assessment (23). The EIRRCs collect information on new cases and report them to the national registry (22).

According to the law, a child is deemed to have Taiwan nationality only when at least one of the parents has Taiwan nationality. Therefore, all the registered cases born to immigrant mothers have a father with Taiwan nationality. According to the Statistics of Birth Reporting System, the majority of immigrant mothers (about 90%) are from China, Vietnam, and Indonesia (15).

### Data Collection

We obtained data on the number of new (incident) cases from the Department of Statistics of Ministry of Health and Welfare. EIRRCs only register those cases who have not attended the primary schools, generally under 7 years of age in Taiwan (22), but the Ministry of Health and Welfare did not provide the data broken down by age. Therefore, we analyzed the data using all children under 7 years of age as one single group.

We obtained data on the number of newborns born to immigrant mothers from the Health Promotion Administration of the Ministry of Health and Welfare (15). However, such data were unavailable before 2004, and therefore we have to limit our analyses to the period between 2010, when newborns in 2004 became 6 years old, and 2013, the year that the most update data were provided by the government.

### Data Analysis

We estimated the annual incidence rate of DD by dividing the number of new cases by the number of children in the study population (under 7 years old) in each year. We presented descriptive statistics of newborns as number and percentage by the maternal nationality and used linear regressions to assess the trends over the years.

Furthermore, to estimate the relative risks, we used the incidence rate of DD in children of mothers from Vietnam, whose incidence rate of DD was in the middle amongst the three groups, as the reference. For example, we divided the incidence rate of DD in children of Indonesian mothers by the incidence rate in children of Vietnamese mothers to obtain the relative risk (incidence rate ratio [RR]) for children of Indonesian mothers. For each RR, we calculated its 95% confidence interval (CI) for evaluating its statistical significance. In addition, we used the Chi-square test for trend to evaluate the statistical significance of trends in incidence rates of DD in children born to mothers of China, Vietnam, and Indonesia nationalities in each year.

We conducted all analyses using SAS 9.1 and performed all statistical tests at the two-tailed significance level of 0.05. The study protocol was reviewed and approved by the Institution Review Board of Ditmanson Medical Foundation Chiayi Christian Hospital (No. 104016).

## Results

### The Number and Proportion of Newborns from Immigrant Mothers

From 2004 to 2013, the number of newborns given birth by immigrant mothers decreased from 29,956 in 2004 to 13,457 in 2013, with a decreasing trend over time (*p* < 0.01) (Table 1). Likewise, the proportion of the newborns from immigrant mothers decreased from 13.8% of the total newborns in 2004 to 6.9% in 2013, showing a decreasing time trend (*p* < 0.01).

**Table 1:** The number and proportion of newborns by nationality of immigrant mothers.

**Year**	**Total (%)** ^**a**^	**China** ^**b**^	**Vietnam**	**Indonesia**	**Other nationalities**
		***N* (%)** ^**c**^	***N* (%)** ^**c**^	***N* (%)** ^**c**^	***N* (%)** ^**c**^
2004	29,956 (13.8)	11,513 (38.4)	13,323 (44.5)	2,808 (9.4)	2,312 (7.7)
2005	27,542 (13.3)	10,142 (36.8)	12,892 (46.8)	2,282 (8.3)	2,226 (8.1)
2006	24,690 (12.0)	10,441 (42.3)	10,143 (41.1)	1,928 (7.8)	2,178 (8.8)
2007	21,420 (10.5)	10,037 (46.9)	7,752 (36.2)	1,720 (8.0)	1,911 (8.9)
2008	19,389 (9.9)	9,631 (49.7)	6,506 (33.6)	1,478 (7.6)	1,774 (9.1)
2009	17,038 (8.9)	8,827 (51.8)	5,344 (31.4)	1,270 (7.5)	1,597 (9.4)
2010	14,603 (8.8)	7,973 (54.6)	4,068 (27.9)	1,111 (7.6)	1,451 (9.9)
2011	15,312 (7.7)	8,658 (56.5)	4,100 (26.8)	1,075 (7.0)	1,479 (9.7)
2012	17,229 (7.3)	9,752 (56.6)	4,570 (26.5)	1,175 (6.8)	1,732 (10.1)
2013	13,457 (6.9)	7,512 (55.8)	3,426 (25.5)	980 (7.3)	1,539 (11.4)

a *The newborns from immigrant mothers accounted for proportion of the total newborns*.

b *Include Hong Kong and Macao*.

c *The proportion of newborns from mothers of a given nationality in total number of newborns by immigrant mothers*.

In term of the nationalities of immigrant mothers, the top three was China, Vietnam, and Indonesia, and they contributed about 90% of the newborns by immigrant mothers in the study period. Although the number of newborns decreased in all three groups of maternal nationality, the proportion of newborns from mother of China nationality had an increasing trend over the years (*p* < 0.01) and reached the top rank in 2006. On the other hand, mothers of Vietnam nationality had the largest proportion of newborns in 2004 and 2005, and proportion became the second in 2006, with a decreasing trend thereafter (*p* < 0.01). The proportion of newborns from mothers of Indonesia nationality remained the third over the years but also had a decreasing trend (*p* < 0.01) (Table 1).

### The Number of Cases and Incidence of Developmental Delays by Maternal Nationality

From 2010 to 2013, 4,604 new cases of DD were registered in children of immigrant mothers, including 1,833 (39.8%) born to mothers from China, 1,922 (41.7%) born to mothers from Vietnam, 495 (10.8%) born to mothers from Indonesia, and 354 born to mothers from other countries (Table 2). The annual incidence rate of DD in children under 7 years old ranged from 6.7 to 7.2 per 1,000 in children born to mothers of China nationality, from 9.6 to 11.0 per 1,000 in children born to mothers of Vietnam nationality, and from 11.1 to 12.5 per 1,000 in children born to mothers of Indonesia nationality (Table 2).

**Table 2:** The number of newly reported case of developmental delays by maternal nationality.

**Year**	**Total**	**China** ^**a**^	**Vietnam**	**Indonesia**	**Other nationalities**
		***N* (%)**	***N* (%)**	***N* (%)**	***N* (%)**
2010	1,321	482 (36.5)	576 (43.6)	152 (11.5)	111 (8.4)
2011	1,153	443 (38.4)	497 (43.1)	125 (10.8)	88 (7.6)
2012	1,100	460 (41.8)	454 (41.3)	108 (9.8)	78 (7.1)
2013	1,030	448 (43.5)	395 (38.3)	110 (10.7)	77 (7.5)

a *Include Hong Kong and Macao*.

### Comparison of Incidence Rates among Children Born to Mother of China, Vietnam, and Indonesia

In comparison with children born to mothers of Vietnam nationality, the incidence rate of DD was lower in children born to mothers of China nationality in every year, and the RRs ranged from 0.65 to 0.73 (all with *p* < 0.05) (Table 3) with an overall RR of 0.69 (95% CI: 0.65–0.73, *p* < 0.05).

**Table 3:** The incidence of developmental delays by maternal nationality (Vietnam as the reference).

**Year**	**Estimated population**			**Incidence rate (per 1,000)**			**Rate ratio [95% Confidence interval]**			
	**China**	**Vietnam**	**Indonesia**	**China**	**Vietnam**	**Indonesia**	**China**	**Vietnam**	**Indonesia**	***p*** ^**a**^
2010	68,564	60,028	12,597	7.0	9.6	12.1	0.73 [0.65, 0.83]^*^	1.00	1.26 [1.05, 1.50]^*^	<0.001
2011	65,709	50,805	10,864	6.7	9.8	11.5	0.69 [0.61, 0.78]^*^	1.00	1.18 [0.97, 1.43]	<0.001
2012	65,319	42,483	9,757	7.0	10.7	11.1	0.66 [0.58, 0.75]^*^	1.00	1.04 [0.84, 1.28]	<0.001
2013	62,390	35,766	8,809	7.2	11.0	12.5	0.65 [0.57, 0.74]^*^	1.00	1.13 [0.92, 1.39]	<0.001

a *For the trend test from China, Vietnam, to Indonesia*.

* *p < 0.05*.

Likewise, in comparison with children born to mothers of Vietnam nationality, the incidence rates of DD were higher in children born to mothers of Indonesia nationality in every year, and the RRs ranged from 1.04 to 1.26 (Table 3). While the RRs in individual years reached statistical significance in 2010 only, the overall RR (1.16) reached statistical significance (95% CI: 1.05–1.28, *p* < 0.05).

The incidence rates of DD among children born to mother of China, Vietnam, and Indonesia showed an increasing trend in each year (*p* < 0.01 for the Chi-square test for trend in all years).

## Discussion

Our study found the incidence rate of DD was the highest in children of mothers from Indonesia, followed by those of mothers from Vietnam, and then those of mothers from China. The results are compatible with our speculation that children born to immigrant mothers with closer language and culture backgrounds are less likely to have DD. Amongst the three, the official language of China is the same as Taiwan, and the two countries shared similar culture such as religion and mothers' knowledge of child development and child rearing (12, 16, 17). Although Vietnam and Taiwan do not have the same official language, Vietnam is more heavily influenced by the Han culture, which is the mainstream culture in Taiwan, than Indonesia. Moreover, Buddhism is the major religion in both Taiwan and Vietnam. Indonesia and Taiwan also have different official languages. Comparing with Vietnam, Indonesia is less influenced by the Han culture, and Islam is the major religion (16, 17, 24). Therefore, China is the closest to Taiwan in terms of language and culture, followed by Vietnam, and then Indonesia. In fact, a study comparing social values and child raising behaviors among Taiwanese, Vietnamese, and Indonesian found that Taiwanese share a greater degree of value similarity with the Vietnamese than with Indonesian, especially on issues related to religious belief and characteristics of child raising (24).

An interview study of Indonesian and Mainland Chinese immigrant brides in Taiwan found that Indonesian brides tend to be socially excluded because of the huge differences in language and culture, while Mainland Chinese brides adapted much better in the Taiwanese society (16). In another study, the same researchers interviewed Vietnamese and Mainland Chinese brides in Taiwan and found that Vietnamese brides were more afflicted by economic and cultural exclusion than Mainland Chinese brides (17). Accordingly, they argued that because the original language and culture (including religion) of the Vietnamese brides are more heavily influenced by Chinese language and culture than those of the Indonesian brides, they fit better to Taiwanese society. The trends in adaptation of the immigrant brides observed by these studies are compatible with the trends in the incidence of DD observed in our study.

Language is a barrier to social adaptation of foreign spouses (25). Language barrier hinders proper expressions of feeling and emotions and thus may affect marriage, which is the most important inter-personal relationship to foreign spouses. In addition, it limits their social interactions with other people and makes it difficult for them to emerge into the community. Language barrier also reduces the opportunities of employment and thus leads to economic difficulties. The difference in language between Taiwan and Mainland China is small, and therefore, spouses from Mainland China have better social adaptation than spouses of Southeast Asia in Taiwan (16, 17, 26). We believe the better social adaptation of foreign spouses contribute to the lower incidence of DD in their children. Language is also a barrier in parenting (27). Many Taiwanese families do not allow a mother of foreign nationality to educate children in her native language. Therefore, we believe the lower incidence rate of DD in children born to mothers of China nationality is most likely attributable to the smaller language barrier (7).

When we used children born to mothers of China nationality as the reference, the average increase in the incidence of DD in those who were born to mothers of Vietnam nationality was around 47% over the 4-year period. In contrast to that, using children born to mothers of Vietnam nationality as the reference, we found the average increase in the incidence of DD in those who were born to mothers of Indonesia nationality was around 15% over the 4-year period. Therefore, the difference in language of the mothers seemed to have a larger effect than that of the difference in culture background. However, scientific literature on this issue is limited, and further studies are needed to evaluate this speculation.

One of the major limitations of the current study is that the government does not provide data on individuals, and so we were unable to adjust for potential confounders such as genetic factors, the maternal education level, and family socioeconomic status. Nonetheless, using “immigrant mother” and “Taiwan” as key words to search literature indexed by PubMed, we found one study with such data on immigrant mothers from the three countries we studied. In that study, a random sample of 1,827 immigrant mothers in Taipei, the capital city of Taiwan located in northern Taiwan, showed that mothers from China had the largest proportion with an educational level at college or higher, followed by those from Indonesia, and then those from Vietnam (28). The sequence does not correlate with that in the incidence of DD in the children observed in our study. Likewise, immigrant mothers from China had the highest dispensable income, followed by those from Indonesia, and then those from Vietnam (28). In addition, a study in southern Taiwan recruited 94 immigrant mothers from China, Vietnam, and Southeast Asian, and according to the statistics, most of those who were from Southeast Asian would be from Indonesia (4). Similar patterns were observed in that study; namely, immigrant mothers from China had the largest proportion with a high educational level, followed by those from Southeast Asian, and then those from Vietnam, while immigrant mothers from China had the largest proportion with a monthly income more than 20,000 New Taiwan Dollars, followed by those from Southeast Asian, and then those from Vietnam. Therefore, differences in the incidence of DD observed in our study were not likely attributable to differences in maternal education level or income.

In addition, the government does not provide data on specific types of DD, we can only study DD as a whole. DD may be underreported in Taiwan, as in many other countries (19). If the proportion of underreporting was higher in children born to mothers of China nationality than that in children born to mothers of other nationalities, it would be a contributing factor for the lower relative risk. However, because of the smaller language barriers, mothers of China nationality can obtain medical and social resources more easily if their children have DD, and therefore underreporting is less likely than that in children born to mothers of Vietnam or Indonesia.

In comparison with previous studies, our study covers the entire nation and generates national estimates, which are rarely reported. In addition, the study population is large and with more than one thousand new cases every year, which enable us to generate stable epidemiological data. Although incidence data are generally more useful than prevalence data in identifying risk factors, the vast majority of previous studies on DD used prevalence data, because incidence data are more difficult to obtain in most cases (19). In this study, we included new cases only and thus used incidence data. Furthermore, the data collection covered a 4-year period, not just one as in most previous nationwide studies, and therefore the results of analysis are more reliable.

## Conclusion

Our study found that children born to immigrant mothers of nationalities with closer language and culture backgrounds had lower incidence of DD. While maternal language seemed to have a stronger association with the incidence of DD among the children than maternal culture background, further studies are needed to evaluate this observation. Nonetheless, these findings support the important role of mothers' language and culture backgrounds in the development of children and cast some light on the prevention and management of DD in young children born to immigrant mothers. Screening on young children born to immigrant mothers with different language or culture backgrounds may identify patients of DD early and thus facilitate early intervention to minimize the impacts of the disorder (29). If further studies with data on individuals confirm our findings, measures to improve immigrant mothers' knowledge of the language and culture habit of the new country, such as education programs or information leaflets, may be taken to reduce their children's risk for developing DD.

## Data Availability Statement

The original contributions presented in the study are included in the article/supplementary material, further inquiries can be directed to the corresponding authors.

## Ethics Statement

The study protocol was reviewed and approved by the Institution Review Board of Ditmanson Medical Foundation Chiayi Christian Hospital (No. 104016).

## Author Contributions

Y-CT and H-RG conceived and designed the study and wrote the paper. D-CL and H-RG collected and analyzed the data. All authors contributed to and have approved the final manuscript.

## Conflict of Interest

The authors declare that the research was conducted in the absence of any commercial or financial relationships that could be construed as a potential conflict of interest.

## Publisher's Note

All claims expressed in this article are solely those of the authors and do not necessarily represent those of their affiliated organizations, or those of the publisher, the editors and the reviewers. Any product that may be evaluated in this article, or claim that may be made by its manufacturer, is not guaranteed or endorsed by the publisher.

## Abbreviations

CI: confidence interval
DD: developmental delays
EIRRC: Early Intervention Reporting and Referral Centers
RR: relative risk (incidence rate ratio).
